# Meeting of the Governors and General Council

**Published:** 1915-12-25

**Authors:** 


### KING EDWARD'S HOSPITAL FUND FOR LONDON.

### Meeting of the Governors and General Council.

A MEETING of the Governors and General Council of King Edward's Hospital Fund for London, for the purpose of awarding grants to the hospitals, convalescent homes, and consumption sanatoria for the present year, was held on December 16 at St. James's Palace, the Speaker of the House of Commons being in the chair.

Lord Revelstoke (the Honorary Treasurer), in reporting that the amount received for general purposes by the Fund to December 11, after payment of expenses, was £124,575, said that it was satisfactory to notice, especially during the difficult times which the financial world had been passing through in the last eighteen months, that the income from investments for 1915 exceeded that for 1914 by about £14,000.

Sir William Collins, in making the annual statement on behalf of the League of Mercy, said the League was happily in a position, at the close of the current year, again to contribute £14,000 to the Fund, as in 1913 and 1914, thus making a total of £230,000 contributed by the League to the Fund since the foundation of the former in 1899.

Sir William Church (the Chairman of the Distribution Committee) presented the report of that Committee (see p. 281). Mr. Frederick M. Fry (Hon. Secretary) presented the list of awards (see p. 282).

Dr. Edwin Freshfield (the Chairman of the Convalescent Homes Committee) presented the report of that Committee (see p. iv), and Mr. John G. Griffiths (Hon. Secretary) presented the list of awards (p. iv).

### The Speaker's Address.

The Speaker of the House of Commons, in moving the adoption of the report and awards, said: My Lords and Gentlemen,—In the continued absence of the Duke of Teck on military duty, I rise on behalf of the Governors to move the adoption of the report. First of all, I have the honour to read the following letter, which His Majesty has been graciously pleased to address to the Presiding Governor, through Lord Stamfordham.

"Buckingham Palace,

"December 15, 1915.

"DEAR SIR,—I am commanded by the King to ask you to convey to the Governors and General Council of King Edward's Hospital Fund his congratulations on the maintenance of the distribution at the same amount as last year.

"His Majesty is interested to see that the total amount distributed by the Fund since its foundation, nineteen years ago, now exceeds £2,000,000—a convincing proof of the success of the scheme inaugurated by his late Majesty King Edward.

"His Majesty trusts that the Fund will continue to receive the generous support of the public, and will thus be enabled effectively to assist the hospitals of London in carrying on their beneficent work during these times of difficulty.

"Yours very faithfully,

"STAMFORDHAM.

"To the Presiding Governor,

"King Edward's Hospital Fund for London."

A LANDMARK IN THE FUND'S HISTORY.

The present distribution is in a sense a landmark in the history of the Fund, owing to the fact that it will make the total amount distributed since the foundation of the Fund a little over £2,000,000, the exact total, in nineteen years, being £2,070,416 8s. 5d. The first million was reached in 1909, when the Fund had been in existence thirteen years. With these figures before us, we may fairly claim that the Fund has succeeded in rendering (to quote the words of the Act of Incorporation) efficient aid and support to the hospitals of London.

This is the second distribution of the Fund that has taken place under war conditions, and I need hardly say that very important questions have had to be considered in fixing the amount to be distributed and in settling the details of the various awards. In both connections we have to look before and after, taking into account not only the present but the future, and to some extent also the past. With regard to the income of the Fund we know what our present position is. Subscriptions and donations have been on the whole very fairly maintained, thanks partly to the loyal support of those who were already contributors, and partly to the welcome help of new subscribers who realise the need of maintaining the Fund's distribution through the period of stress.

THE CONTRIBUTION FROM THE LEAGUE OF MERCY.

It is particularly gratifying that the League of Mercy should have been able to make the same contribution as last year, exactly one-tenth part of the total distributed. Legacies show a considerable reduction, but this, fortunately, is to some extent balanced by the additional income received from the investments representing the late Sir Julius Wernher's munificent bequest to the capital of the Fund. The result is that we are enabled to make the same distribution as last year. Though this is a smaller total than in the years before the war, the demands in aid of capital expenditure are still so much less than in 1913 that the grants to maintenance are actually larger. We are, therefore, still able to achieve the object aimed at by our financial policy in the good years of the past—namely, that the hospitals should, as far as possible, continue to receive from the Fund, in relation to their individual needs, the assistance for which they have become accustomed to look.

To what extent that assistance can be continued in full measure in the future, only the future can show. All we can say is that the needs of the future are constantly present to the minds of the committees which have charge of the resources of the Fund—the Executive Committee, which sees to the collection of the funds and recommends the amount to be distributed, and the Finance Committee, which has the care of the investments. Whatever can be done by those committees to safeguard the future we feel sure has been, and is being, done.

PRESENT DIFFICULTIES AND FUTURE NEEDS.

But this Fund exists not for its own sake but for the sake of the hospitals, and their needs, future as well as present, are also the subject of the most careful consideration of the committees charged with the recommendations as to the total, and the details, of the dis-

THE KING'S FUND.

tribution. The future needs of the hospitals are, of course, a matter of speculation. It is obvious that there is a danger of bad times ahead. Prices have risen and are rising. There is a scarcity in all ranks of hospital service, medical and lay, from the most skilled to the least skilled. All this adds to their expenditure. There are ever-increasing demands for other purposes upon the purses of their regular supporters. On the other hand, there is the spirit of mutual assistance and of sacrifice which has, so far, produced a volume of voluntary giving far above the normal. Whether the share of this volume, which has been given to the hospitals, has kept pace with their growing needs, and, if so, how long it will continue to do so, it is, perhaps, too early to say.

The Hospitals' Present Needs.

As regards the present needs of the hospitals, to meet which the present distribution of the Fund is designed, this again is, much more than in normal times, a matter of estimate—almost of conjecture. Of necessity, therefore, the Fund is compelled to go by the evidence of the past. The audited accounts, the accounts to December 31, 1914—these are firm ground, and the only firm ground, for deciding the financial considerations involved in the present distribution. These show the effect on the finances of the hospitals of all the factors that were the uncertain subjects of estimate a year ago—the increase of expenditure, the effect of the war upon income, the additional work of placing at the disposal of the Government the magnificent resources of the voluntary hospitals for the treatment of the wounded.

At the present moment the results of these same influences during 1915 are again matters of estimate, but at the next distribution they will in their turn be reflected in the accounts, and if in any instances the Fund's grant, made on accounts nearly a year old, proves to be larger or smaller than present circumstances would justify, this will itself be shown in the year's accounts, and will be an important factor in determining next year's grant. Thus justice is done, and in no other way could it be done.

Apart from these questions of finance and the general policy to be adopted under war conditions, there is but little in the events of the year to require mention. The Executive Committee have kept themselves informed as far as possible on the various points on which the national requirements in connection with the war affect the hospitals, and have always been ready to make representations or obtain information whenever it appeared that such action would be agreeable to the hospitals and could be more readily undertaken by the Fund than by the hospitals separately.

Losses by Death.

To the loss which the Fund sustained in the spring through the death of Lord Rothschild, Honorary Treasurer, and Chairman of the Finance Committee, reference was made at the annual meeting last May. It is with great regret that we learned of the death, in July last, of Mr. William Latham, whose resignation of the Chairmanship of the Convalescent Homes Committee, which he had held for eighteen years, was announced a year ago.

The Governors' Thanks.

To all the members of the Council, members of Committees, honorary officers, and visitors of the Fund, the Governors desire to tender their very best thanks. With so many members, medical and lay, absent on war service, the work of the Fund makes larger demands on those who are available, themselves often busy men, rendered still more busy by other activities arising out of the war. I now beg to move the adoption of the Reports of the Distribution and Convalescent Homes Committees.

Mr. Walter Morrison having seconded the motion, the adoption of the Reports was then put and carried unanimously.

Cheques Posted.

On the motion of Lord Iveagh, seconded by the Lord Mayor (Sir Charles Wakefield), it was resolved : "That the cheques for the grants should be posted on Tuesday, December 21, except in the case of grants for the reservation of beds in consumption sanatoria during 1916, which should be posted on January 1."

Sir Ernest Cassel's Gift.

Sir Savile Crossley (Hon. Secretary) said it was his pleasant duty to announce that the Right Hon. Sir Ernest Cassel proposed to give again, as in 1911, £50,000 to hospitals, this time in the form of 4½ per Cent. War Loan Stock. Of this, £28,000 would, if accepted by the Governors and General Council, be given to the Fund to be distributed among the hospitals, convalescent homes, and consumption sanatoria receiving grants from the Fund this year, in amounts equal to one-fifth of the grants made by the Fund. The remaining £22,000 had been allocated to certain hospitals and kindred institutions in London and the country. The distribution would be effected as soon as practically possible, and the list with details would be published (see p. 284).

On the motion of the Speaker, it was resolved that the Fund gladly accepts the very munificent offer of Sir Ernest Cassel, and asks Sir Savile Crossley to convey to Sir Ernest Cassel the thanks of the Governors and General Council for his renewed benevolence to the Fund and for the great assistance he had thus rendered to the hospitals of London.

The Council for 1916 with New Members.

The Speaker of the House of Commons then said : My Lords and Gentlemen,—I have to announce that there are three vacancies on the Council, and the Governors have decided to add the following names : The Hon. Nathaniel Charles Rothschild, Sir Walter Lawrence, Bt., Sir Francis Champneys, Bt. I will now ask the Hon. Secretaries to read the list of members of the Council, members of Committees, and honorary officers for the year 1916.

The list was then read by Mr. Frederick Fry (Hon. Secretary).

The Annual Resolutions.

The Speaker continued : No alterations are necessary in the usual resolutions delegating powers to the various Committees. It is customary to have these resolutions approved at this meeting so that the meeting of the new Council called in January to pass them need only be formal.

On the motion of Lord Knutsford, seconded by Sir William Bennett, a resolution was approved delegating powers to the Finance Committee, Distribution Committee, and Convalescent Homes Committee.

On the motion of the President of the Royal College of Physicians (Dr. Frederick Taylor), seconded by Sir John Tweedy, a resolution was approved delegating to the Distribution Committee the power to draw upon the

**THE KING'S FUND.**

grants set aside for the amalgamation of throat hospitals. On the motion of Mr. Algernon H. Mills, seconded by Sir Henry Burdett, a resolution was approved delegating powers to the Executive Committee.

### Vote of Thanks to Speaker.

Lord Mersey, in moving a vote of thanks to the Speaker of the House of Commons for presiding, said they were all very grateful for the great interest he had taken for many years past in this Fund, and were very much obliged to him for the clear and comprehensive statement he had made with reference to the present condition of the Fund and its future prospects.

The resolution was seconded by Sir Horace Marshall, and carried unanimously.

The Speaker, in acknowledging the vote of thanks, said : I am very much obliged for this vote of thanks. It is always a great pleasure to me to be able to be present on these occasions. I feel that I can do little on behalf of the Fund, but what little I can do I am only too glad to do when opportunity offers, and I am glad to place my services at your disposal at any time.

